# Dexmedetomidine Moderate Sedation Versus General Anesthesia on the Diagnostic Yield of Endobronchial Ultrasound-Guided Transbronchial Needle Aspiration: A Randomized Controlled Study

**DOI:** 10.5812/aapm-146646

**Published:** 2024-07-10

**Authors:** Mohammad Fouad Algyar, Mohamed Torky, Ahmed Mohamed Ibrahim, Mohamed Abdelbadie, Mhmoud A Almohasseb, Saad Ahmed Moharam, Taysser Abdelraheem

**Affiliations:** 1 Anesthesiology, Surgical Intensive Care and Pain Medicine, Faculty of Medicine, Kafrelsheikh University, Kafrelsheikh, Egypt; 2 Chest Department, Faculty of Medicine, Tanta University, Tanta, Egypt; 3 Anesthesiology, Surgical Intensive Care and Pain Medicine Department, Faculty of Medicine, Tanta University, Tanta, Egypt; 4 Anesthesiology, Surgical ICU and Pain Management, Faculty of Medicine, Beni-Suef University, Beni-Suef, Egypt

**Keywords:** Dexmedetomidine, Endobronchial Ultrasound-Guided Transbronchial Needle Aspiration, General Anesthesia, Sedation

## Abstract

**Background:**

One of the factors that affect the diagnostic yield of endobronchial ultrasound-guided transbronchial needle aspiration (EBUS-TBNA) is the level of sedation.

**Objectives:**

Therefore, we aimed to compare dexmedetomidine (DEX) as moderate sedation (MS) versus general anesthesia (GA) on the diagnostic yield of EBUS-TBNA.

**Methods:**

This randomized open-label controlled trial was carried out on 70 patients older than 18 years of age, classified as American Society of Anesthesiologists (ASA) II or III, and scheduled for EBUS-TBNA. Patients were randomly allocated into two equal groups. Group D received 1 μg/kg fentanyl 2 minutes before induction with a 1 μg/kg infusion of DEX for 10 minutes, then maintenance with 0.5 - 1 μg/kg/h aiming for a Ramsey Sedation Scale of 4 - 5 while preserving hemodynamics. Group GA received 1 μg/kg fentanyl, 2 mg/kg propofol, and 0.5 mg/kg atracurium (then 0.1 mg/kg every 20 minutes).

**Results:**

Group D had a significantly higher rate of recalling the procedure (P = 0.005) and a lower rate of shortness of breath compared to group GA (P = 0.038). Intraoperative heart rate measurements at baseline were not significantly different between groups but were significantly lower at 5 min, 10 min, 15 min, 20 min, and at the end of surgery in group D compared to group GA (P < 0.05). Intraoperative mean arterial blood pressure measurements at baseline, 5 min, 10 min, 15 min, 20 min, and at the end of surgery were not significantly different between groups. Recovery time was significantly shorter in group D compared to group GA (P < 0.001).

**Conclusions:**

Compared to GA, MS with DEX showed a comparable diagnostic yield with faster recovery time and better patient satisfaction, as evidenced by a willingness to repeat procedures when needed and less shortness of breath in EBUS-TBNA.

## 1. Background

Endobronchial ultrasonography (EBUS) to guide transbronchial needle aspiration (EBUS-TBNA) is a minimally invasive, highly effective method for obtaining tissue samples from peribronchial, mediastinal, and pulmonary masses for pathological analysis (1, 2). All accessible lymph nodes (LNs) are sampled during EBUS staging (3).

Two main approaches are used during EBUS: Moderate sedation (MS) and general anesthesia (GA) (4). Moderate sedation is characterized by the patient's ability to maintain spontaneous ventilation and respond purposefully to verbal or tactile stimuli, with no interventions required to maintain a patent airway (5). The sedation method employed during endobronchial ultrasound-guided transbronchial needle aspiration (EBUS-TBNA) can impact diagnostic precision, safety, and patient comfort (6, 7).

A combination of an opiate analgesic and a benzodiazepine with amnestic effects is commonly used to achieve MS (8). On the other hand, dexmedetomidine (DEX) is a potential alternative sedative due to its high affinity as an adrenergic agonist of the alpha-2 receptor (9). Dexmedetomidine does not cause respiratory depression, cognitive impairment, or decreased patient compliance (10). This is because DEX acts on the alpha-2 receptors in the locus coeruleus and does not affect GABA receptors/cerebral cortex, unlike other sedatives like midazolam and propofol (11).

Studies evaluating moderate to deep sedation versus GA for EBUS operations have shown conflicting findings. Some studies reported lower patient satisfaction with MS using DEX (12, 13), while others showed comparable results (14).

## 2. Objectives

Therefore, we established this study to compare DEX as MS versus GA on the diagnostic yield of EBUS-TBNA. The primary outcome was the diagnostic yield of EBUS-TBNA. The secondary outcomes were hemodynamics, procedure time, recovery time from anesthesia, and safety.

## 3. Methods

This randomized open-label controlled trial included seventy patients, aged over 18 years, of both sexes, with American Society of Anesthesiologists (ASA) classifications of II or III. These patients required EBUS-TBNA for mediastinal staging of lung cancer and had suspected benign or malignant mediastinal or hilar LNs.

This study was conducted after approval by the Faculty of Medicine Ethical Committee, Tanta University Hospitals, Tanta University, Tanta, Egypt (approval code 35719/9/22), and was registered on clinicaltrials.gov (ID: NCT05781035). The study took place from April 2023 to October 2023. Informed written consent was obtained from all patients.

Excluded from the study were patients with a body mass index of 35 or higher, those allergic to any of the sedatives or anesthetics used, pregnant women, and those needing additional procedures during a planned bronchoscopy (such as therapeutic bronchoscopy, navigational bronchoscopy, and endobronchial biopsies).

### 3.1. Randomization and Blindness

Randomization was done utilizing a computer-generated sequence through sealed opaque envelopes in a parallel manner into two groups: Group D, which received MS with DEX, and the GA group, which received GA. The study was open-label due to the different techniques used.

A complete medical history, physical examination, and diagnostic testing were performed on all patients. Following cannula insertion, all cases were premedicated intravenously (IV) with 2 mg of midazolam and were monitored via pulse oximetry, ECG, non-invasive blood pressure, capnography, and a temperature probe.

In group D, patients received 1 μg/kg of fentanyl two minutes before induction with a 1 μg/kg infusion of DEX for 10 minutes, followed by maintenance with 0.5 - 1 μg/kg/h, aiming for a sedation level of 4 - 5 while preserving hemodynamics. The Ramsey Sedation Scale (RSS) was used to assess the depth of sedation (15). This scale divides a patient's level of sedation into six categories ranging from severe agitation to deep coma. The DEX group used a nasal cannula to provide oxygen while the mouthpiece supplied EBUS. Patients were kept under spontaneous breathing.

In group GA, GA was induced with 2 mg/kg of propofol IV and 1.0 μg/kg of fentanyl IV. For endotracheal intubation enhancement, 0.5 mg/kg of atracurium was given IV, followed by 0.1 mg/kg of atracurium IV every 20 minutes. Isoflurane 1 - 1.5% was used to maintain anesthesia. End-tidal CO_2_ was kept between 30 and 35 mmHg using mechanical ventilation (respiratory rate 10 to 14 breaths per minute, tidal volume 6 to 8 mL/kg, inspiratory-to-expiratory ratio 1 to 2, positive end-expiratory pressure 5 cm H_2_O).

In both groups, if the patient's heart rate (HR) or mean arterial pressure (MAP) increased by more than 20% from their baseline values, they received bolus doses of 1 μg/kg fentanyl IV. Bradycardia (HR < 50 beats/min) was treated with 0.01 mg/kg atropine. Hypotension (MAP ≤ 65 mmHg or a reduction in MAP of > 20% from the preoperative baseline value) was treated with 5 - 10 mg IV ephedrine. All operations were carried out by the same surgical team.

### 3.2. Endobronchial Ultrasound-Guided Transbronchial Needle Aspiration Technique

The convex transducer (7.5 MHz) (Pentax - EB-1970Uk 2.0 mm working channel) was included in a flexible ultrasonic bronchoscope. An ultrasound console was used to adjust the images. After identifying the lesion of interest, a 22-gauge needle was used for transbronchial punctures. Each lymph node was punctured with the needle at least four times. Three aspirates were taken at each lymph node station for lung cancer staging, as the diagnostic yield reached a plateau after three passes, with only small increases in yield after four or more passes (16). The aspirated material was placed in a container with a preservative solution before being processed into a cytoproct and stained for cytological analysis. Cytologists were blinded to the group assignments.

One bronchologist with more than four years of experience conducted the EBUS-TBNA. The hemodynamic parameters of the patients (HR and MAP) were recorded every five minutes until the end of the procedure.

Following each procedure, the number of LNs sampled per patient, the number of biopsies per LN, and the size of the LN were recorded. "Procedure time" was defined as the duration between bronchoscope insertion and removal from the airway. The diagnostic yield of EBUS was calculated by counting the number of patients for whom EBUS-TBNA established a definitive diagnosis. Diagnoses included total diagnostic yield, malignancy, granulomatous disease, and reactive inflammatory LNs.

Aldrete's score was used to evaluate the recovery time, identifying time 0 as the moment the patient was moved from the operating table to the stretcher and evaluating the score every 15 minutes until a score of 8 was reached (15).

The patients were continuously monitored in the recovery room until they had fully recovered. Patient satisfaction and tolerance of the endobronchial ultrasound procedure were assessed before discharge using a specialized questionnaire (17). Additionally, a questionnaire was used to assess the difficulties experienced by bronchoscopists and anesthesiologists (6). All questionnaires were validated in Arabic.

Analyses of hemodynamic parameters and the incidence of EBUS-TBNA complications, as well as complications from anesthesia, were used to determine the procedure's safety. Pulmonary auscultation was used to diagnose laryngospasm and bronchospasm and to decide whether the patient required emergency care or bronchodilators.

Complications from EBUS-TBNA and sedation/anesthesia included cardiovascular events (such as bleeding, pneumothorax, mediastinitis, or mediastinal abscess) and respiratory events (such as hypoxemia, defined as partial pressure of SpO_2_ below 90% for more than 30 seconds requiring intervention such as a non-rebreathing mask, "bagging," or mechanical ventilation). Other noted complications included inadequate sedation despite the maximum allowed dosages of sedatives, arrhythmia necessitating antiarrhythmic drugs, and severe coughing that prevented the procedure from being completed. All complications were recorded.

### 3.3. Sample Size Justification

G.power 3.1.9.2 (Universitat Kiel, Germany) was used for the sample size calculation. Based on a 0.05 α error and 80% power of the study, the expected diagnostic yield was 68% versus 95%, according to previous studies (6, 17). To account for potential dropouts, an additional six cases were included. Therefore, 35 patients were allocated to each group.

### 3.4. Statistical Analysis

The data were analyzed using IBM's SPSS v26 (Chicago, Illinois, USA). An unpaired Student's *t*-test was used to analyze quantitative variables, with results presented as mean and standard deviation (SD). When applicable, the chi-square test or Fisher's exact test was used to examine qualitative variables, provided as frequencies and percentages. A P-value < 0.05 with two tails was considered statistically significant.

## 4. Results

**Figure 1.**
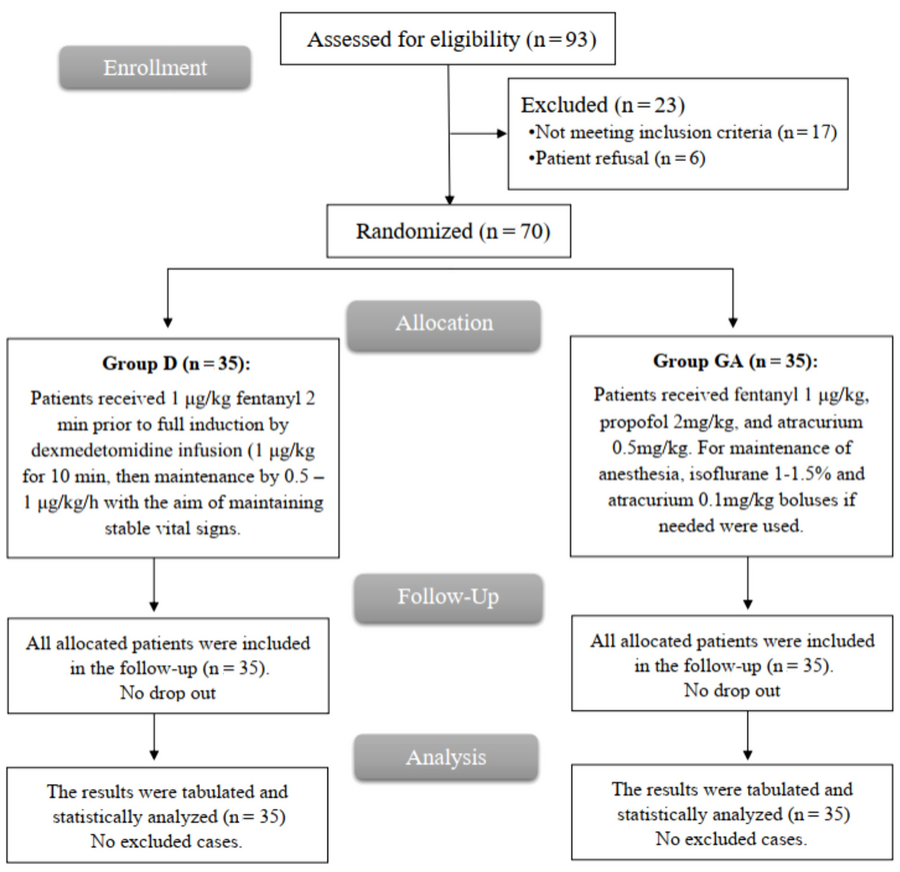
CONSORT flowchart of the enrolled patients

After screening for study eligibility, 93 patients were eligible; however, 17 did not meet the requirements, and 6 declined to participate. The remaining patients were equally divided into two groups (35 per group). All patients were followed up and analyzed statistically (Figure 1). 

Demographic data and medical history were comparable between both groups (Table 1). 

**Table 1.** Demographic Data and Medical History of the Studied Groups ^a^

Variables	Group D (n = 35)	Group GA (n = 35)	P-Value
**Age (y)**	53.69 ± 15.2	50.31 ± 13.8	0.335
**Sex**			
Male	27 (77.14)	25 (71.43)	0.584
Female	8 (22.86)	10 (28.57)	
**Weight (kg)**	74.66 ± 10.44	75.77 ± 9.89	0.648
**Height (m)**	1.69 ± 0.08	1.7 ± 0.05	0.274
**BMI (kg/m** ^**2**^ **)**	26.42 ± 4.27	26.14 ± 3.35	0.759
**ASA physical status**			
II	13 (37.14)	11 (31.43)	0.615
III	22 (62.86)	24 (68.57)	
**Medical history**			
DM	16 (45.71)	15 (42.86)	0.810
Hypertension	17 (48.57)	19 (54.29)	0.632
Smoking	19 (54.29)	25 (71.43)	0.138

Abbreviations: ASA, American Society of Anesthesiologists; DM, diabetes mellitus; GA, general anesthesia; D, DEX.

^a^ Values are expressed as mean ± SD or No. (%).

**Figure 2.**
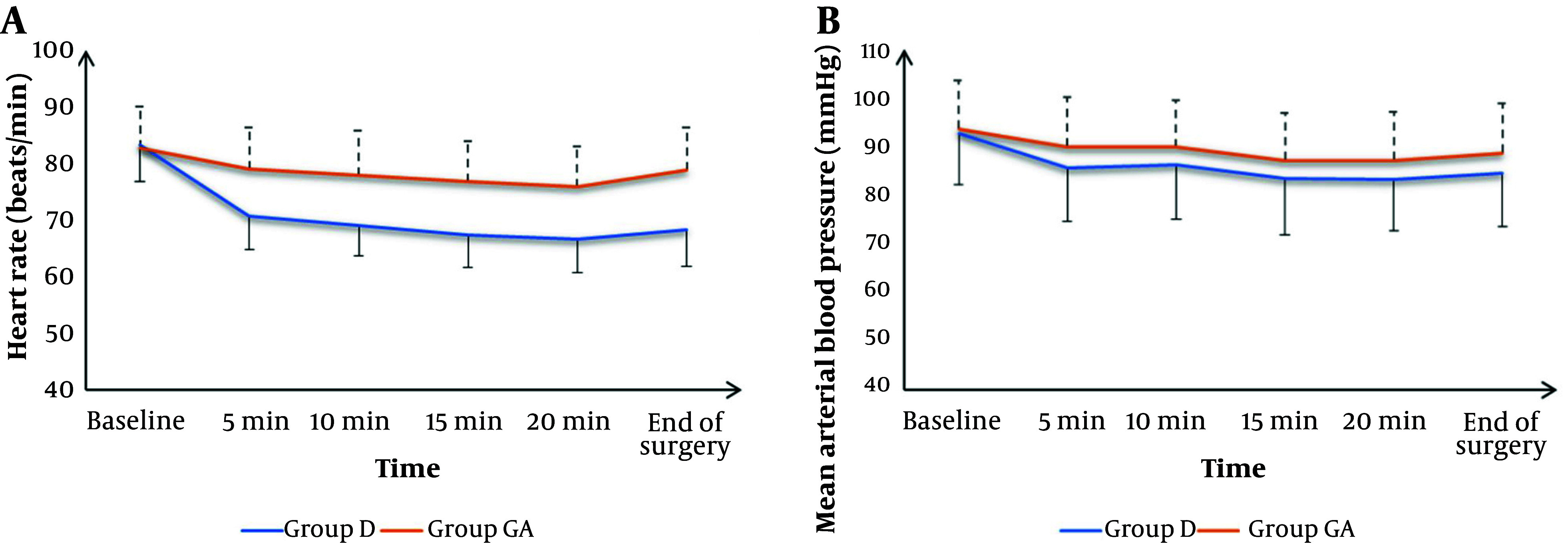
A, heart rate; and B, mean arterial blood pressure changes of the studied groups. Data are shown as mean ± SD.

Intraoperative HR measurements at baseline were not significantly different between groups but were significantly lower at 5 min, 10 min, 15 min, 20 min, and at the end of surgery in group D compared to group GA (P < 0.05). Intraoperative mean arterial blood pressure measurements at baseline, 5 min, 10 min, 15 min, 20 min, and at the end were not significantly different between groups (Figure 2). 

The total diagnostic yield was 25 (71.43%) patients in group D and 27 (77.14%) in group GA. Malignancy was diagnosed in 19 (54.29%) patients in group D and 17 (48.57%) patients in group GA. Granulomatous disease was diagnosed in 4 (11.43%) patients in group D and 6 (17.14%) patients in group GA. Reactive inflammatory LNs were found in 2 (5.71%) patients in group D and 4 (11.43%) patients in group GA. Intraoperative fentanyl consumption, procedure time, diagnostic yield, EBUS-related complications, complications (hypertension, hypoxemia, excessive cough, arrhythmia, and excessive secretions), number of LNs, number of biopsies per lymph node, and size of LNs were comparable between both groups. Recovery time was significantly shorter in group D than in group GA (P < 0.001). Inadequate sedation occurred in 2 (5.71%) patients in group D (Table 2). 

**Table 2.** Procedure Data and Characteristics of Lymph Nodes of the Studied Groups ^a^

Variables	Group D (n = 35)	Group GA (n = 35)	P-Value
**Intraoperative fentanyl consumption (µg/kg)**	75.14 ± 10.95	76.86 ± 9.63	0.489
**Procedure time (min)**	32.86 ± 5.98	31.86 ± 5.95	0.485
**Recovery time (min)**	7.66 ± 1.43	11.06 ± 1.33	< 0.001
**Diagnostic yield**			
Total	25 (71.43)	27 (77.14)	0.584
Malignancy	19 (54.29)	17 (48.57)	0.576
Granulomatous disease	4 (11.43)	6 (17.14)	
Reactive inflammatory LNs	2 (5.71)	4 (11.43)	
**EBUS-related complications**	20 (57.14)	19 (54.29)	0.810
**Complications**			
Hypertension	6 (17.14)	5 (14.29)	0.743
Hypoxemia	5 (14.29)	3 (8.57)	0.452
Excessive cough	4 (11.43)	1 (2.86)	0.164
Arrhythmia	1 (2.86)	5 (14.29)	0.088
Excessive secretions	2 (5.71)	5 (14.29)	0.232
Inadequate sedation	2 (5.71)	-	-
**Number of lymph node **	2.89 ± 1.23	3.23 ± 1.73	0.343
**Biopsies per lymph node**	4.29 ± 1.15	4.06 ± 1.03	0.384
**Lymph node size (mm/short axis)**	15.34 ± 8.23	14.43 ± 5.56	0.589

Abbreviations: EBUS, endobronchial ultrasonography; GA, general anesthesia; D, DEX; LNs, lymph nodes.

^a^ Values are expressed as mean ± SD or No. (%).

Group D had a significantly higher rate of recalling the procedure (P = 0.005) and a lower rate of shortness of breath compared to group GA (P = 0.038) (Table 3). 

**Table 3.** Patient Satisfaction and Tolerance of Endobronchial Ultrasound Procedure Questionnaire of the Studied Groups

Variables	Group D (n = 35)	Group GA (n = 35)	P-Value
**Would you undergo this procedure again, in the future?**			
Definitely not	0 (0)	2 (5.71)	0.431
Probably not	1 (2.86)	0 (0)	
Unsure	2 (5.71)	3 (8.57)	
Probably would	6 (17.14)	8 (22.86)	
Definitely would	26 (74.29)	22 (62.86)	
**How much do you recall the procedure?**			
None	15 (42.86)	27 (77.14)	0.005
Small amount	11 (31.43)	7 (20)	
Significantly	9 (25.71)	1 (2.86)	
**How would you rate your cough?**			
None	13 (37.14)	14 (40)	0.881
Small	17 (48.57)	15 (42.86)	
Significantly	5 (14.29)	6 (17.14)	
**How would you rate your sore throat?**			
None	22 (62.86)	17 (48.57)	0.434
Small	11 (31.43)	14 (40)	
Significantly	2 (5.71)	4 (11.43)	
**How would you rate your chest pain?**			
None	34 (97.14)	32 (91.43)	0.614
Small	1 (2.86)	3 (8.57)	
**How would you rate your shortness of breath?**			
None	31 (88.57)	23 (65.71)	0.038
Small	4 (11.43)	8 (22.86)	
Significant	0 (0.0)	4 (11.43)	

Abbreviations: GA, general anesthesia; D, DEX.

^a^ Values are expressed as mean ± SD or No. (%).

According to the bronchoscopist and anesthesiologist questionnaire, group D had significantly higher difficulties compared to group GA (Table 4). 

**Table 4.** Bronchoscopist and Anesthesiologist Questionnaire of the Studied Groups ^a^

Variables	Group D (n = 35)	Group GA (n = 35)	P-Value
**Bronchoscopist questionnaire**			
**In which of the following steps did you feel difficulty?**			0.002
EBUS introduction	4 (11.43)	0 (0)	
Target ultrasound identification/recognition	1 (2.86)	0 (0)	
TBNA	9 (25.71)	2 (5.71)	
Other	1 (2.86)	1 (2.86)	
None	21 (60)	28 (80)	
**Difficulty reason**			< 0.001
Patient’s movement	3 (8.57)	0 (0)	
Cough	11 (31.43)	1 (2.86)	
Anatomic factors related to the target lesions	6 (17.14)	1 (2.86)	
None	15 (42.86)	33 (94.29)	
**Anaesthesiologist questionnaire**			
**In which of the following steps did you feel difficulty?**			0.044
Induction	2 (5.71)	0 (0)	
Maintenance	9 (25.71)	2 (5.71)	
Recovery	2 (5.71)	4 (11.43)	
None	22 (62.86)	29 (82.86)	
**Which factors contributed to the above difficulties?**			0.355
Difficult airway	5 (14.29)	1 (2.86)	
Laryngospasm	6 (17.14)	4 (11.43)	
Hemodynamic alterations	2 (5.71)	1 (2.86)	
Other	1 (2.86)	1 (2.86)	
None	21 (60)	28 (80)	

Abbreviations: EBUS, endobronchial ultrasonography; GA, general anesthesia; TBNA, transbronchial needle aspiration; D, DEX; TBNA, transbronchial needle aspiration.

^a^ Values are expressed as mean ± SD or No. (%).

## 5. Discussion

The bronchoscopist's choice of sedation for EBUS-TBNA is still a crucial consideration. Procedure results, patient satisfaction, and financial factors play roles in determining the optimal method of sedation. The available data attempting to address this issue are limited (12, 18).

Under GA and neuromuscular blocking, the bronchoscopist may achieve the best possible procedure conditions, which in turn improves the quality of the surgery and the patient's comfort (12, 19). The drawbacks of GA include the need for an artificial airway, the risks associated with mechanical ventilation, prolonged procedure time, and increased costs (20, 21).

Therefore, sedation offers several benefits over GA, including a lower hemodynamic effect, shorter recovery period, and avoidance of the need for an artificial airway and controlled ventilation (22). Guidelines from the American College of Chest Physicians (ACCP) and the EBUS community encourage using both moderate and deep sedation (23).

The major findings of our study showed that sedation with DEX or GA was effective and safe for patients undergoing EBUS-TBNA, with similar diagnostic yields and no major complications in either group. The GA group showed better hemodynamics, lower difficulty sensation during procedures according to the bronchoscopist questionnaire, and better sedation according to the anesthesiologist questionnaire. However, sedation with DEX was more favorable due to the lower recovery time and incidence of shortness of breath, making patients more likely to repeat the procedure if needed.

In line with our results, Casal et al. (17) demonstrated that MS was associated with a shorter recovery time than GA. They also found that the diagnostic yield and sensitivity of EBUS-TBNA were not significantly impacted by either GA or MS. Ost et al. (24) also reported that neither MS nor GA affects the diagnostic yield of EBUS-TBNA.

Additionally, Casal et al. (17) showed that patients in the MS group more often recalled the procedure (P < 0.001) and experienced lower shortness of breath post-EBUS-TBNA (P = 0.016). Yarmus et al. (14) conducted retrospective research on EBUS and found that the diagnostic yield was 66% with MS.

Contrasting results were obtained by Lin et al. (25), who showed that patients who received DEX during EBUS-TBNA had a reduced ability to perceive the procedure and a decreased intention to undergo the operation again due to the lesser sedation.

Moreover, Fernandes et al. (6) showed that the bronchoscopists' and anesthesiologists' assessments of the procedure's difficulty were comparable between the GA and MS groups. There was no difference in sedation depth between the two groups. It is generally agreed that EBUS-TBNA is a safe technique. This difference may be due to different MS techniques, as patients received combinations of midazolam, propofol, and/or alfentanil/fentanyl, plus local oropharynx and larynx anesthesia with 2% lidocaine.

Boujaoude et al. (12) showed that for patients undergoing EBUS-TBNA, there was no significant difference between MS and Monitored Anesthesia Care (sedation provided and monitored by an anesthesiologist) in terms of diagnostic yield (92.9 vs. 91.9%), operation time, or major complication rate, although MS may have minor adverse effects such as hypotension and desaturation.

The study had certain limitations: Firstly, it was conducted at a single center, which may limit the generalizability of the findings. Additionally, the investigators responsible for administering sedation were not blinded to the patients' titration regimen, which could introduce bias. The use of different doses and additives to DEX, as well as including a diverse range of procedures, is recommended in future research.

### 5.1. Conclusions

Compared to GA, MS with DEX showed a comparable diagnostic yield with faster recovery time and better patient satisfaction, as evidenced by a willingness to repeat procedures when needed and less shortness of breath during EBUS-TBNA.

## Data Availability

The dataset presented in the study is available on request from the corresponding author during submission or after publication.

## Clinical Trial Registration Code:

Registration on clinical trials.gov (ID: NCT05781035) from April 2023 to October 2023.
